# Perihepatic Biloma in a Non-cirrhotic Patient After Transjugular Intrahepatic Portosystemic Shunt (TIPS)

**DOI:** 10.7759/cureus.23399

**Published:** 2022-03-22

**Authors:** Faisal Mehmood, Amina Khalid, Shalom Frager

**Affiliations:** 1 Hospital Medicine, Montefiore Medical Center, Bronx, USA; 2 Hospital Medicine, Albert Einstein College of Medicine, Bronx, USA; 3 Transplant Hepatology, Albert Einstein College of Medicine, Bronx, USA

**Keywords:** oral contraceptive pill (ocp), transjugular intrahepatic portosystemic shunt (tips), biloma, biliary injury, portal thrombosis

## Abstract

Biloma is an intrahepatic or extrahepatic collection of bile within the abdominal cavity. It can occur spontaneously, or as a result of trauma to the biliary tree. The clinical presentation can be variable and non-specific. Early diagnosis is crucial given the high mortality rate. Diagnostic modalities include abdominal ultrasound, hepatobiliary scintigraphy, computerized tomography (CT), and magnetic resonance imaging (MRI). Treatment options include interventional radiology (IR)-guided drainage, endoscopic drainage, or surgical drainage with a bile leak repair. We report a case of a middle-aged non-cirrhotic patient who presented with abdominal pain and was noted to have extensive portal vein thrombosis. She underwent transjugular intrahepatic portosystemic shunt (TIPS) with thrombectomy and the hospital course was complicated by elevated liver enzymes and found to have intrahepatic biloma requiring IR-guided drainage.

## Introduction

Transjugular intrahepatic portosystemic shunt (TIPS) is an effective therapeutic option and is minimally invasive procedure for managing portal hypertension complications. It has more than 90% success rate [1]. The advantage of TIPS is offset by some serious complications. The incidence of these fatal complications is reported to be less than 5%. The most commonly reported complications of TIPS include hepatic encephalopathy, liver failure, infection at TIPS site, renal failure, shunt stenosis or occlusion, TIPS associated hemolysis, and stent migration to the portal vein or the right atrium [2-4]. It is uncommon to have biliary complications after TIPS. However, any delay to identify and treat these complications can have life-threatening consequences. We report a case of a non-cirrhotic female patient who underwent TIPS for portal vein thrombosis and the post-procedural course was complicated by perihepatic biloma.

This study was previously presented as an abstract at the ACG Annual Scientific Meeting in Las Vegas on October 24, 2021.

## Case presentation

**Figure 1:**
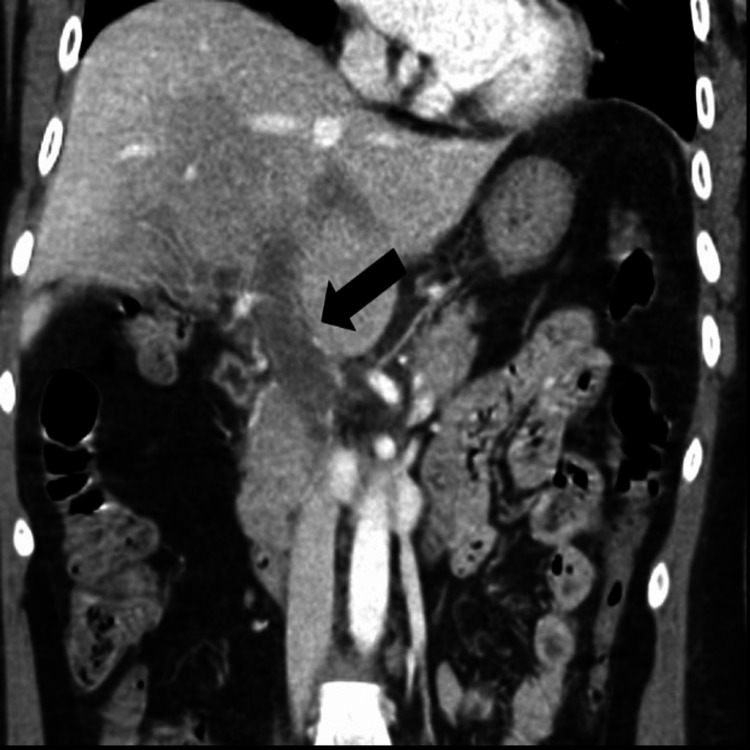
CT abdomen and pelvis, coronal image, shows a large thrombus in the main portal vein (arrow) extending into the right and left portal venous branches.

A 40-year-old woman presented with epigastric abdominal pain for five days. She did not have any significant medical history. She reported taking oral contraceptive pills (OCPs) for one month before the presentation. She denied smoking, alcohol, or using herbal supplements. She denied any family history of clotting disorder or cancer. She had epigastric tenderness on examination. Laboratory investigations revealed stable normocytic anemia (hemoglobin {Hgb}: 10 g/L) on admission labs. Other blood test results were normal including kidney function, liver enzymes, prothrombin time, and international normalized ratio. CT scan of the abdomen showed a large thrombus in the main portal vein extending into the right and left portal venous branches (Figure 1).

**Figure 2:**
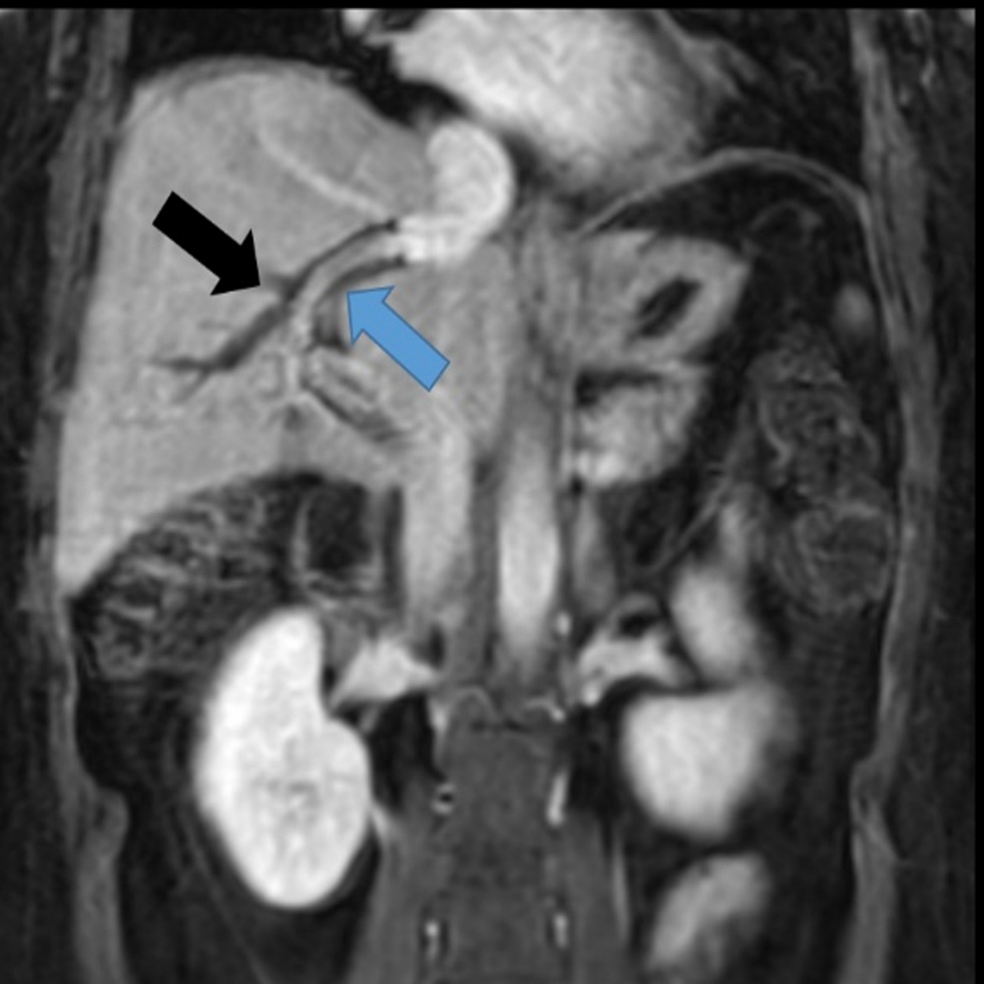
MRI abdomen, coronal post-contrast image, shows patent TIPS (blue arrow) and persistent complete thrombosis of all intrahepatic portal venous branches (black arrow). TIPS: transjugular intrahepatic portosystemic shunt

The patient was started on heparin drip. Due to the extensive nature of the clot and its extension into portal vein branches, it was decided to pursue TIPS with thrombectomy. A few days later, she was noted to have the elevation of liver enzymes with alkaline phosphatase of 510 U/L, aspartate aminotransferase (AST) of 149 U/L, alanine transaminase (ALT) of 150 U/L, and a normal bilirubin level. MRI of the abdomen showed patent TIPS and persistent complete thrombosis of all intrahepatic portal venous branches requiring a repeat interventional radiology (IR) guided thrombolysis (Figure 2). However, liver enzymes continued to rise after a few days with alkaline phosphatase of 552 U/L, AST of 293 U/L, and ALT of 324 U/L. She was also found to have a low-grade fever and lab investigation showed mild leukocytosis (15 K/uL) but denied any abdominal pain.

**Figure 3:**
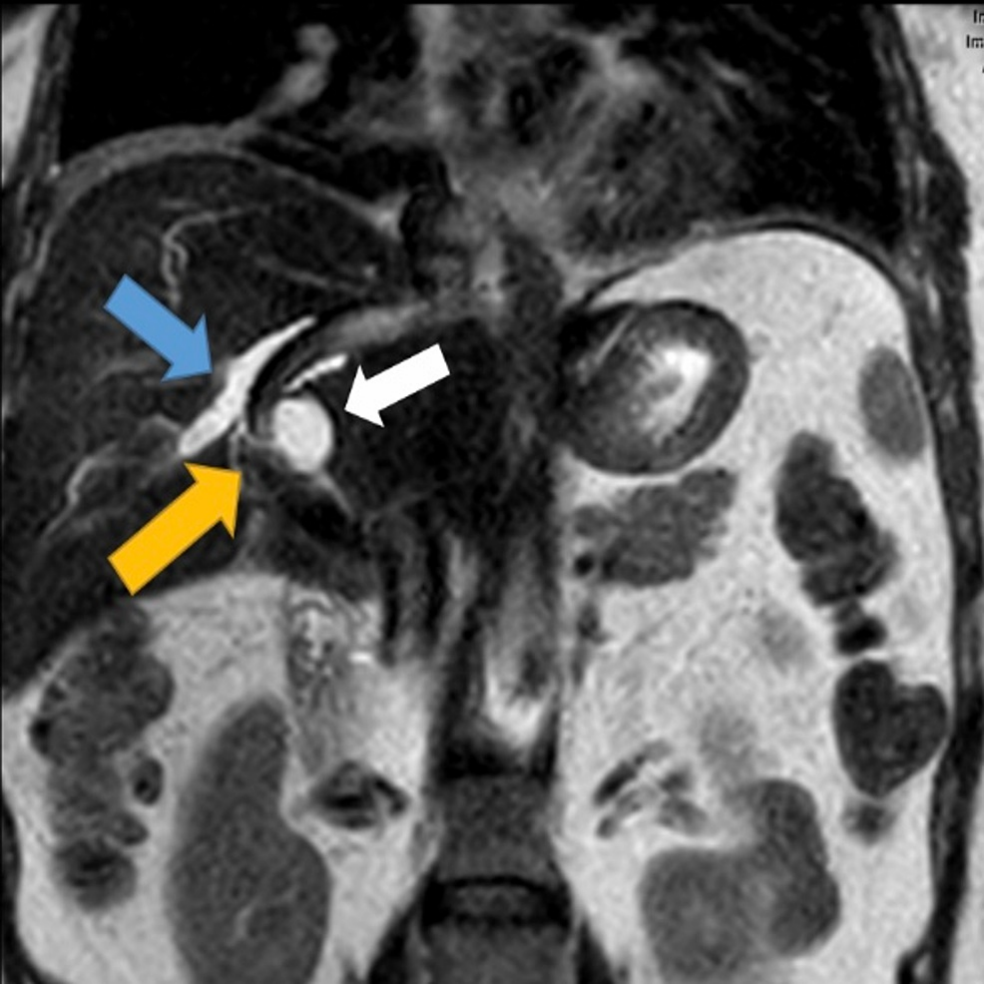
MRI abdomen, coronal T2-weighted image, shows a new intrahepatic biloma (white arrow) abutting a portion of the TIPS (yellow arrow) with upstream intrahepatic biliary ductal dilatation (blue arrow). TIPS: transjugular intrahepatic portosystemic shunt

**Figure 4:**
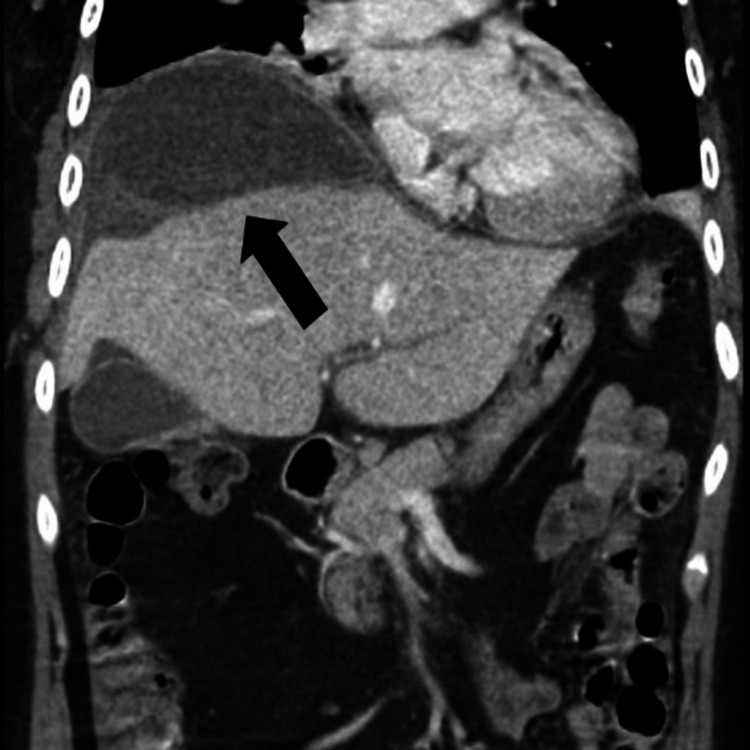
CT abdomen and pelvis, coronal image, shows a new peri-hepatic collection surrounding the hepatic dome (arrow).

A repeat MRI of the abdomen was done and showed intrahepatic biloma at the mid aspect of TIPS with upstream intrahepatic biliary ductal dilatation (Figure 3). It was decided to pursue IR-guided percutaneous cholecystostomy. However, she continued to have a fever after the cholecystostomy. A follow-up CT scan was done and showed a 13 cm complex fluid collection at the dome of the liver (Figure 4).

**Figure 5:**
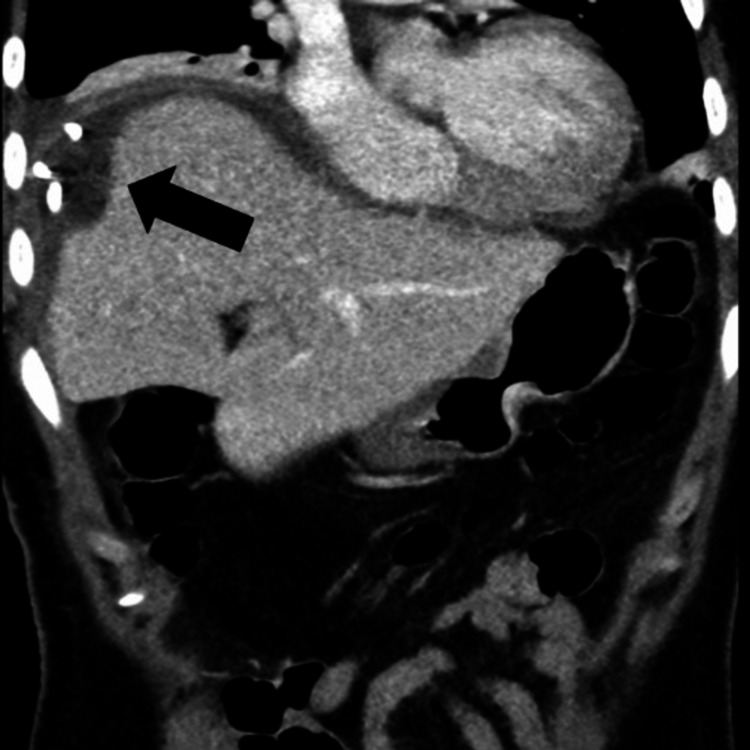
CT abdomen and pelvis, coronal image, shows interval placement of a percutaneous drainage catheter and decrease in size of the peri-hepatic collection (arrow).

She underwent IR-guided drainage of the new collection with the removal of 150 cc bilious fluid, and fluid cultures were sent. A catheter was placed in the hepatic dome collection. She received intravenous cefoxitin for seven days with an interval decrease in the collection size (Figure 5). Fluid cultures did not grow any organisms.

Hypercoagulable workup including protein C and S deficiency, antithrombin III (ATIII), JAK2 mutation, factor V Leiden mutation, prothrombin gene mutation, beta-2 glycoprotein, anticardiolipin, lupus anticoagulant, and paroxysmal nocturnal hemoglobinuria (PNH) flow cytometry were unremarkable.

After receiving a few days of antibiotics, fever resolved, transaminases normalized, and had slightly elevated alkaline phosphatase (288 U/L). The biliary drainage catheter was removed and she was discharged in stable condition. MRI abdomen on eight months follow-up demonstrated resolution of intrahepatic bilomas. She did not have any symptoms and liver tests were completely normal.

## Discussion

The incidence of biloma is estimated to be 0.3-2%, most often affecting older adults in the sixth and seventh decades of life. There is no gender preponderance suggesting that it can affect males and females equally [5,6]. The most common location of bilomas is intrahepatic or in the right subphrenic or subhepatic spaces [5,7]. Bilomas can occur spontaneously, or as a result of trauma to the biliary tree. The most common cause of spontaneous biloma is choledocholithiasis. Other causes include bile duct tumors, liver infarction, abdominal trauma, and iatrogenic bile duct injury because of cholecystectomy, percutaneous catheter drainage, transhepatic cholangiogram, endoscopic retrograde cholangiopancreatography (ERCP), liver biopsy, radiofrequency ablation, and transcatheter arterial chemoembolization [7]. It is uncommon to have a severe bile duct injury after TIPS and is reported to be present in less than 1% of cases [8]. However, in some cases, a biloma may form after TIPS and is amendable to either percutaneous drainage of the fluid cavity or endoscopic placement of a biliary stent to drain the biliary system [2]. Bilomas have a high mortality rate unless promptly diagnosed and treated [7]. Differential diagnoses include hepatic cyst and hematoma, liver abscess, loculated ascites, and post-operative fluid collections (including seroma and lymphocele).

The clinical manifestations of biloma are variable and non-specific, including abdominal pain, fever, jaundice, and cholestatic pattern of liver injury if there is associated biliary obstruction from biloma. The presentation can be life-threatening if the patient has sepsis from secondary bacterial infection and abscess formation or bile peritonitis. Diagnosis is based on clinical and radiological findings with ultrasound, hepatic scintigraphy, CT scan, or MRI findings. Fluid aspiration with analysis can be a helpful tool to confirm the diagnosis. Conservative approach can be used if the size of biloma is less than 4 cm. However, drainage is the mainstay of treatment for large size bilomas along with antibiotics if infected. Follow-up imaging should be done to monitor the size in patients requiring conservative management or requiring drainage to ensure resolution.

It is very rare to have a thrombus in portal vasculature in patients without underlying liver disease. There are few reported cases of portal vein thrombosis among patients after long-term use of OCPs but none of these were severe enough requiring TIPS and thrombolysis [9-12]. Our patient was taking OCP for a short period and developed extensive portal vein thrombosis requiring TIPS and thrombectomy. She developed biloma, a rare complication after TIPS requiring IR-guided drainage. However, she did not report symptoms concerning biloma and only had a low-grade fever and was noted to have elevated alkaline phosphatase and transaminases. Most of the reported complications are related to iatrogenic injury to the portal vein, hepatic artery, or bile duct, leading to fistula formation [13-17]. In rare instances, complications after TIPS are so severe that patients required liver transplantation [18-20].

## Conclusions

Although biloma formation is uncommon after TIPS. Measures must be taken to prevent these complications. Interventional radiologists require a thorough stepwise understanding of TIPS insertion, possible adverse sequela, and technical moves to maximize the safety of the procedure. Controlled needle passage should be practiced along with limited needle puncture to prevent any fistulous communication between biliary or arterial system and portal vein. In case of complication, consider the case thoroughly.

Clinicians should include biloma in the differential diagnosis if a patient continues to have abdominal pain, fever, or elevated liver enzymes after TIPS. A multidisciplinary team of physicians including hepatologists, gastroenterologists, surgeons, interventional radiologists, and infectious disease experts should be used to provide care for these patients. IR-guided percutaneous drainage is an effective treatment option. However, follow-up imaging should be done to monitor the size and resolution.
